# Automatic Recognition of Fetal Facial Standard Plane in Ultrasound Image via Fisher Vector

**DOI:** 10.1371/journal.pone.0121838

**Published:** 2015-05-01

**Authors:** Baiying Lei, Ee-Leng Tan, Siping Chen, Liu Zhuo, Shengli Li, Dong Ni, Tianfu Wang

**Affiliations:** 1 Department of Biomedical Engineering, School of Medicine, Shenzhen University, National-Regional Key Technology Engineering Laboratory for Medical Ultrasound, Guangdong Key Laboratory for Biomedical Measurements and Ultrasound Imaging, Nanhai Ave 3688, Shenzhen, Guangdong, P. R. China, 518060; 2 Department of Ultrasound, Affiliated Shenzhen Maternal and Child Healthcare, Hospital of Nanfang Medical University, Shenzhen, China; 3 School of Electrical and Electronic Engineering, Nanyang Technological University, Singapore, 639798, Singapore; University Medical Center Groningen UMCG, NETHERLANDS

## Abstract

Acquisition of the standard plane is the prerequisite of biometric measurement and diagnosis during the ultrasound (US) examination. In this paper, a new algorithm is developed for the automatic recognition of the fetal facial standard planes (FFSPs) such as the axial, coronal, and sagittal planes. Specifically, densely sampled root scale invariant feature transform (RootSIFT) features are extracted and then encoded by Fisher vector (FV). The Fisher network with multi-layer design is also developed to extract spatial information to boost the classification performance. Finally, automatic recognition of the FFSPs is implemented by support vector machine (SVM) classifier based on the stochastic dual coordinate ascent (SDCA) algorithm. Experimental results using our dataset demonstrate that the proposed method achieves an accuracy of 93.27% and a mean average precision (mAP) of 99.19% in recognizing different FFSPs. Furthermore, the comparative analyses reveal the superiority of the proposed method based on FV over the traditional methods.

## Introduction

Due to the relatively low cost, real-time imaging capability, and avoidance of radiation exposure [1–4], ultrasound (US) has been widely used for pregnancy diagnosis. During the US-based diagnosis progress, the clinician first identifies the standard plane by checking the existence of main anatomical structures, and then examines the plane for further diagnosis and interpretation of the fetal growth. The acquisition of standard plane requires substantial experience as well as good knowledge of the human anatomy. Hence, this task is extremely challenging for novices and time consuming even for experienced examiners. Highly accurate automatic recognition of standard plane is not only extremely useful in assisting both experienced and inexperienced examiners, but also beneficial to underprivileged countries since trained imaging experts might be scarce in such countries.

Recently, considerable effort has been devoted to automatic recognition of the standard plane from US images [1, 2, 5–12]. Due to the high similarity between the standard and non-standard planes, high intra-class variations of standard plane caused by various gestational ages, different fetal postures, various scanning orientations, and the presence of speckles and artifacts in US images, automatically recognizing standard planes remains challenging. The first step to address low accuracy in recognition is to find the proper feature representation. State-of-the-art feature representations include single feature (i. e., Haar-type, histogram of gradient (HoG) or scale invariant feature transform (SIFT)) as well as combination of motion, intensity, shape, and edges [2, 13–16]. In recent years, densely sampled SIFT [5, 17–21] has become a promising technique for image representation. Enhancement to the SIFT includes the root scale invariant feature transform (RootSIFT) [22], which not only inherits good property of SIFT such as invariance to scale and rotation, but also improves the recognition performance. Furthermore, it is extremely simple to convert from SIFT to RootSIFT without introducing additional requirements for computation and storage [22]. In view of this, RootSIFT is selected for the feature extraction in the proposed standard plane recognition algorithm.

In order to enhance the recognition performance and efficiency, dense features are usually encoded prior to transformation into a histogram of occurrence. Currently, the most popular encoding methods for learning and recognition are locally linear embedding (LLE) [23], vector of locally aggregated descriptor (VLAD) [20], and Fisher vector (FV) [17, 24], which essentially are extensions of the bag of visual words (BoVW) [25]. In the last decade, deep learning has attracted considerable attention and is applied in a myriad of field due to its powerful discriminative learning ability [26]. The feature statistics especially high-order statistics with the introduction of feature weights outperforms the standard handcrafted representation or binary representation [27]. Moreover, it is known that feature hierarchy with multilayer feature encoding [28] is very effective in exploiting both architecture and feature hierarchy information, which are useful in enhancing classification performance. Inspired by the promising performance of deep learning and feature hierarchy, feature hierarchy with multilayer design is investigated in this work to improve the FFSP recognition performance.

The recognition task is achieved by processing the encoded features using the popular support vector machine (SVM) method since it can obtain global optimal values and overcome the over-fitting problem in classification [29–31]. As shown in [32], stochastic dual coordinate ascent (SDCA) is a novel approach which possesses advantages over traditional stochastic gradient descent (SGD) method [33], such as faster convergence rate and higher efficiency in learning as compared to SGD. In addition, SDCA provides the duality gap value (the difference between primal and dual energy). Therefore, we applied SDCA to effectively address the convex quartic programming optimization problem in SVM.

By exploring the state-of-the-art techniques, we propose an effective solution to automatically recognize the fetal facial standard plane (FFSP) from US images. Our goal is to assist clinicians to automatically localize the FFSP during US examinations. The main contributions of this paper are four-fold. Firstly, feature extraction is based on the densely sampled RootSIFT descriptor and normalization of these features is subsequently performed. Secondly, the extracted features are encoded by FV to incorporate the generative model and higher order difference of the features. Thirdly, learning and recognition of the FFSPs is performed by a SVM classifier based on the SDCA algorithm. Fourthly, the multilayer Fisher network with feature hierarchy is applied to enhance the performance of the proposed method.

## Related Work

Due to the importance of the standard plane from US images for US scanning and diagnosis in clinical practices, automatic localization of standard plane from US images has become a hot topic and attracted a myriad of interest [2, 9, 34]. For example, Carneiro *et al*. [2] proposed to locate the standard planes from 3D US data using probability boosting tree technique and marginal learning space. Rahmatullah *et al*. [9] proposed to automatically detect the standard plane from the 3D US volume. In [34], Cuingnet *et al*. suggested a system to perform fast alignment of fetal head based on 3D US image using random forest and template deformation. Although 3D US is a promising imaging modality for prenatal examination, 2D exam is still the preferred approach due to its better imaging quality, wider availability of 2D scanners, and higher familiarity with experienced users.

More recently, numerous works were proposed to localize standard planes from 2D US images by detecting major anatomical structures [8, 11, 12]. For instance, Zhang *et al*. [8] proposed to intelligently identify and detect the standard plane of the gestational cancer using local context information and cascaded AdaBoost. Rahmatullah *et al*. [11] presented a method to detect anatomical structures from manually extracted abdominal US images by integration of global and local features. This method is semi-automatic and the reported detection accuracy is still inadequate for clinical use and diagnostic purposes. Kwitt *et al*. [12] proposed to localize target structures from US videos by building kernel dynamic texture (KDT) models, which were evaluated based on the US videos acquired from three different phantoms. Since actual patient data is much more complex than phantom data, further investigation is required to evaluate the effectiveness of their proposed method.

Other works reported in [15, 16, 34, 35] are based on object classification method, which are similar to our proposed recognition system. In [15], the low level GIST feature based on Gabor filter was adopted for echocardiogram view classification. Kernel-based SVM classifier was used for an 8-way view classification. The work in [16] utilized the HOG feature and SVM classifier. In [35], a set of novel salient features were located from the edge-filtered motion magnitude images for 2D echocardiogram view classification at scale invariant points. The extracted features are then encoded using local spatial, textural, and kinetic information. A hierarchical feature dictionary was learnt for view classification based on SVM classifier with pyramid matching kernel.

## Methodology

### System Framework

**Fig 1:**
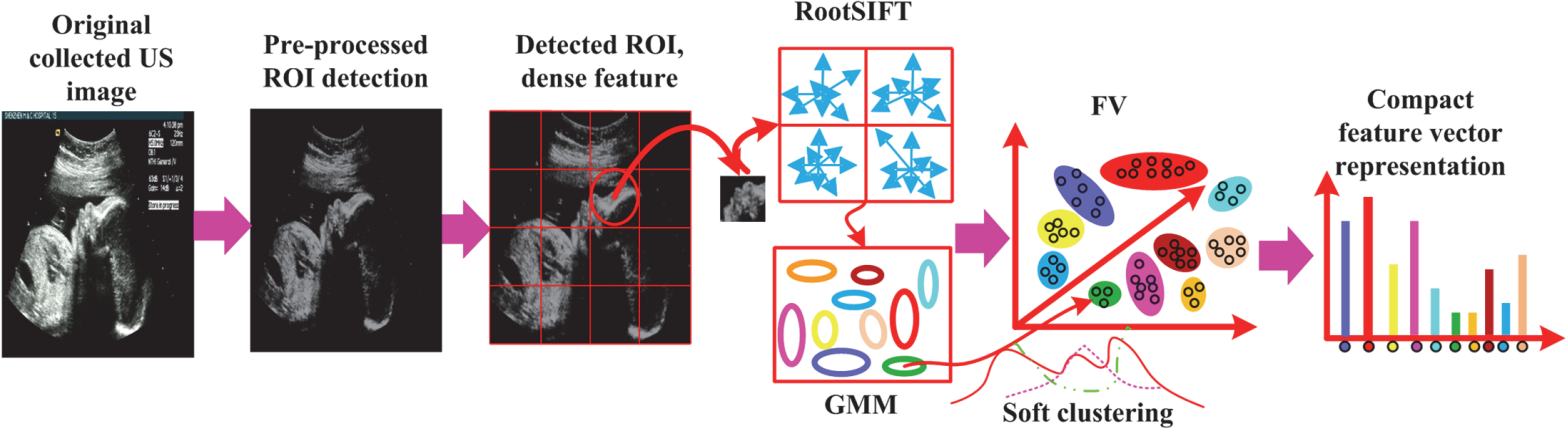
Steps for feature vector extraction. After preprocessing and region of interest (ROI) detection, the root scale invariant feature transform (RootSIFT) is adopted for feature extraction. The extracted features are clustered by Gaussian mixture model (GMM) and then encoded by Fisher vector (FV). The final feature vectors are represented by histogram.

**Fig 2:**
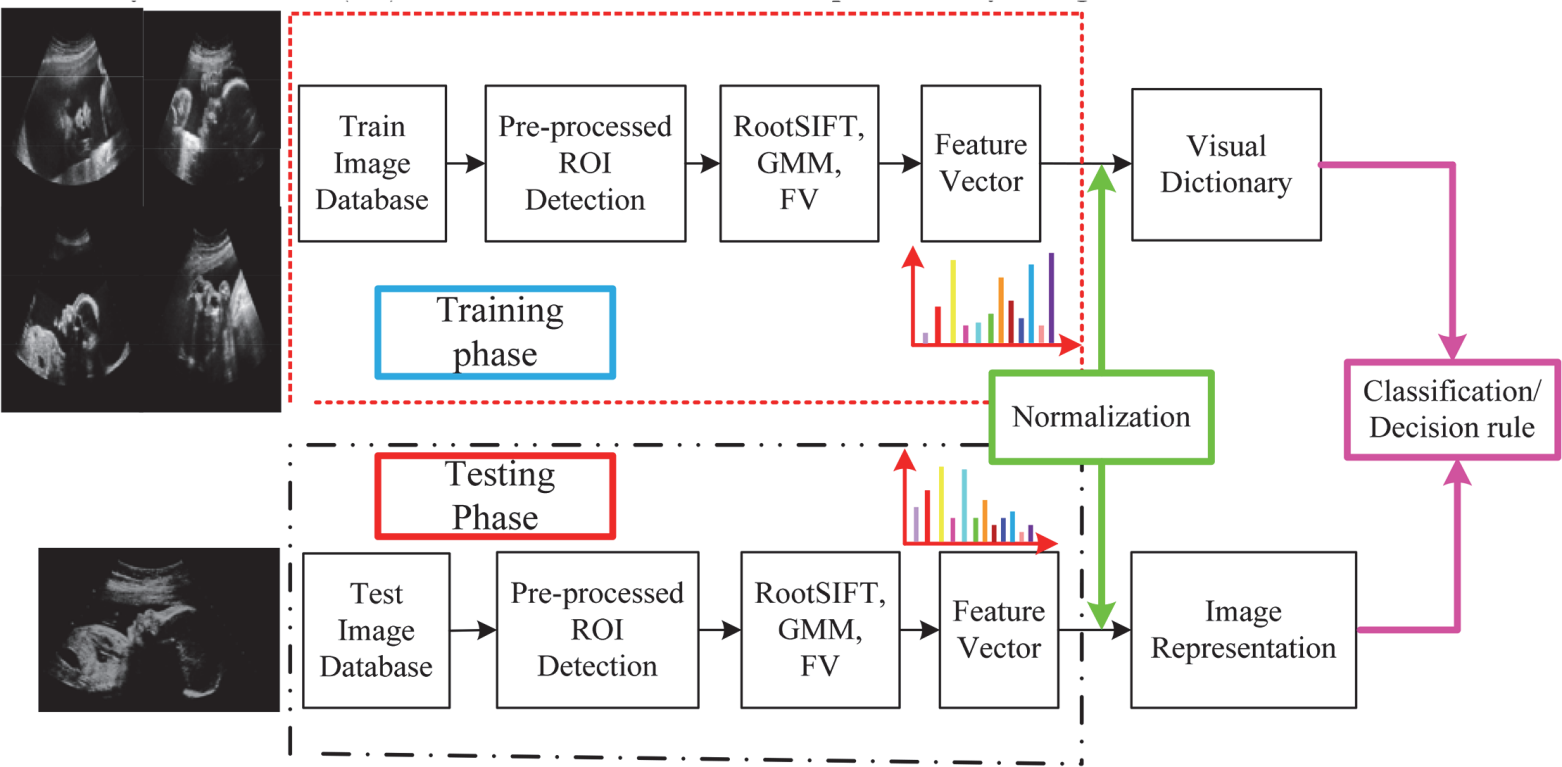
Framework of the proposed fetal facial standard plane (FFSP) recognition system. Images in training and testing phases go through same processing, which comprises root scale invariant feature transform (RootSIFT), Gaussian mixture model (GMM), and Fisher vector (FV). RootSIFT is used for extracting the features, followed by GMM for soft clustering the extracted features. GMM parameters such as mean and covariance are encoded by FV. After encoding, histogram is adopted as the final feature for classification. The recognition decision rule is based on the classification output.

Several pre-processing of the original US images including noise reduction and image enhancement are applied prior to automatic recognition of FFSP to enhance the FFSP recognition. After pre-processing, the regions containing the axial, coronal, and sagittal planes (namely region of interest, ROI) are selected to reduce the search range. The features in the ROI region are extracted using dense sampled RootSIFT and then encoded by FV. The procedure for feature vector construction and representation is shown in Fig 1, and the framework of the proposed FFSP recognition system is illustrated in Fig 2. As shown in the framework, the input US image is partitioned into patches, and each patch is represented by the patch descriptor using component-wise RootSIFT. To reduce the number of descriptor, the input US images are down-sampled to a fixed size *M* (*M* is defined as 480 in our algorithm) without changing the image aspect ratio, and images smaller than *M* × *M* remain unchanged. The feature descriptor extracts the RootSIFT from the downsampled image, and then the Gaussian mixture model (GMM) is applied to the extracted descriptor to generate *k* Gaussians (*k* is set to 80 to trade-off between complexity and efficacy in our algorithm) based on the assumption of a diagonal covariance matrix. After GMM, a set of *k* Gaussians are encoded by FV into a single vector. A histogram is formed by calculating the number of occurrence count of FV representatives. Feature vector is normalized by the feature normalization (i.e., signed power law normalization). Finally, one-versus-rest SVM classifier based on SDCA is employed to determine a predicted class based on the cosine similarity metric.

**Fig 3:**
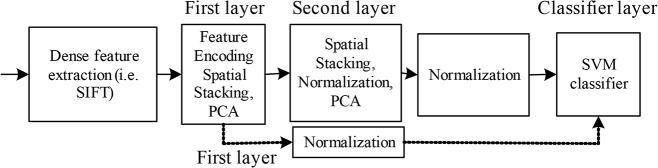
Illustration of multilayer Fisher network with feature hierarchy, where SIFT is scale invariant feature transform, PCA is principle component analysis, and SVM is support vector machine.

Inspired by the promising performance of the popular deep learning method, the multilayer Fisher network architecture is explored to further enhance the FFSP recognition performance. The motivation to design the multi-layer Fisher network with feature hierarchy is that it is able to extract more discriminative information than the single layer Fisher network. Fig 3 illustrates the feature hierarchy with multilayer Fisher networks.

### Feature Extraction

The RootSIFT is selected for feature extraction due to its advantage for non-linear processing and denoted as:

RootSIFT=sqrt(SIFT/sum(SIFT)). 1

Feature extracted by RootSIFT is similar to applying the Hellinger (short for Hel. in this paper) kernel to the original SIFT feature, and hence it outperforms SIFT by kernel method. Hel. or Chi2 distance demonstrates better recognition performance than Euclidean distance. The better performance is attributed to the fact that the Euclidean distance is sensitive to larger values, whereas Hel. distance is often dominated by the smaller values. Hel. distance for *l*_1_ normalized *x* and *y* (*n*-vectors) is computed as:

$$H(x,y)=\sum_{i-1}^{n}\sqrt{x_{i}y_{i}}.$$ 2

Assuming ‖*x*‖_2_ = ‖*y*‖_2_ = 1, Euclidean distance is defined as:

$$d_{E}(x,y)={\left\|x-y\right\|}_{2}^{2}={\left\|x\right\|}_{2}^{2}+{\left\|y\right\|}_{2}^{2}-2x^{T}y=2(1-x^{T}y).$$ 3

By replacing *x* with *x*′ (*x*′ is obtained by the element-wise square root), *x*′ is then *l*_2_ normalized, the Euclidean distance in the feature map space becomes equivalent to the Hel. distance in the original space:

$$x{'}^{T}y'=H(x,y).$$ 4

Using RootSIFT descriptor, the Euclidean distance on SIFT in every step is modified to the Hel. distance with little or no additional requirement on computational cost and storage.

### Multilayer Fisher Vector with Spatial Stacking

**Fig 4:**
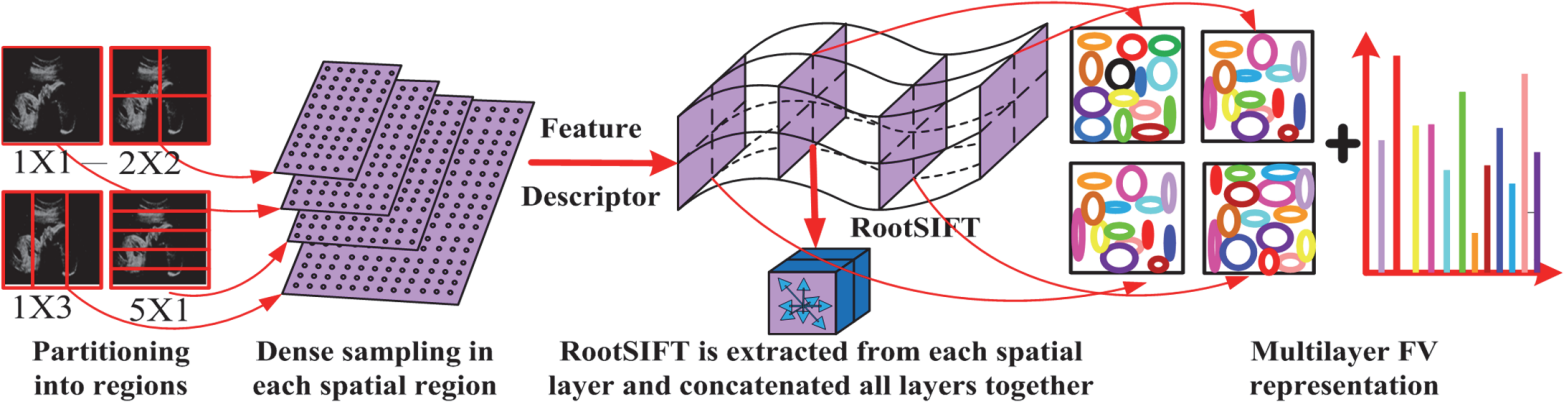
Illustration of dense sampling and spatial stacking. Features are sampled densely in each region. A pyramid model represents the multiple regions. Each layer is spatially stacked and then represented by multilayer Fisher vector (FV).

It is noted that spatial relationships [25] among local appearances play an important role in recognition of the underlying structure of US image. Meanwhile, it has been proved that global or local structure information with multilayer feature hierarchy is effective in improving the descriptive power of the image representation. By incorporating the spatial location of images, spatial pyramid model in [25] achieves better performance compared to BoVW. Spatial pyramid is applied to divide the image into regions to incorporate spatial distribution information. The feature vector extracted from the densely sampled [19] RootSIFT can be stacked with the feature from each FV region to devise the hierarchy of feature. Accordingly, the multilayer feature vectors are built by concatenating all features extracted from every layers and regions. Fig 4 shows the dense sampling and spatial layout in the FFSP recognition task. Dense sampling is integrated with RootSIFT to densely extract features with a multi-resolution grid. Finally, the means and variances of the occurrence of each visual word are concatenated to form the spatial layout of FV, which is the main difference between spatial pyramid model and spatial layout model of FFSP recognition.

### Fisher Vector

**Fig 5:**
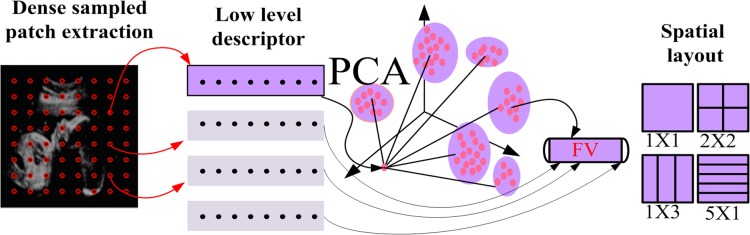
Pipeline of fetal facial standard plane (FFSP) based on Fisher vector (FV). Dense sampled patches are represented by low level descriptor first and then principle component analysis (PCA) is performed on the descriptors. Spatial layout is used to represent different partitions.

Motivated by encouraging results in [17], the generative GMM model in image representation is applied in our system to improve its recognition performance. Fig 5 shows the standard pipeline of FV. Given a codebook learned by the *K*-means: {*μ*_*k*_,*k* = 1,…,*K*}, a set of local descriptors: {*x*_*m*_,*m* = 1,…,*N*}, the steps to extract the feature vector are as follows:

1 Assign neighboring:

$$NN(x_{m}^{})=\underset{\mu_{k}}{\mathrm{argmin}}\left\|x_{m}-\mu_{k}\right\|.$$ 5

2 Compute *v*_*k*_:

$$v_{k}=\sum_{x_{m}:NN(x_{m})=\mu_{k}}x_{m}-\mu_{k}.$$ 6

3 Concatenate *v*_*k*_ and normalize all feature vectors.

For a graphical representation, the dimension of a fixed length vector *v*_*k*_ is dependent on the number of parameters. In order to optimize parameters that better fit the data, higher order statistics (i.e. derivative) are concatenated. A GMM model is built to fit the feature vectors and the derivatives of log-likelihood of the GMM model are encoded by FV. For this approach, the Gaussian means and variances of the first and second order derivatives [17] between dense features and GMM center are computed as:

$$\Phi_{k}^{\left(1\right)}=\frac{1}{N\sqrt{w_{k}}}\sum_{m=1}^{N}\gamma_{m}(k)\left(\frac{x_{m}-\mu_{k}}{\sigma_{k}}\right),$$ 7

$$\Phi_{k}^{\left(2\right)}=\frac{1}{N\sqrt{2w_{k}}}\sum_{m=1}^{N}\gamma_{m}(k)\left(\frac{{\left(x_{m}-\mu_{k}\right)}^{2}}{\sigma_{k}^{2}}-1\right),$$ 8

where {w_*k*_,*μ*_*k*_,*σ*_*k*_} are the GMM mixture weights, means, and diagonal covariance. *γ*_*m*_(*k*) is the soft assignment weight of the *m*-th feature *x*_*m*_ of the *k*-th Gaussian. By concatenating the difference vectors together: $\phi =[\ldots ,\Phi_{1}^{\left(1\right)},\Phi_{1}^{\left(2\right)},\ldots ,\Phi_{k}^{\left(1\right)},\Phi_{k}^{\left(2\right)},\ldots ]$, FV *ϕ* is obtained. The main purpose of the encoding is to discriminate the distribution difference between a specific test image and all fitted training image. FV is soft assigned VLAD with high-order statistics and an essential extension of BoVW. For *D* dimensional feature vector, the main difference between the BoVW and FV can be represented as:

$$\phi_{BoVW}(x_{m})=[0,\ldots ,0,1,0,\ldots ,0],$$ 9

$$\phi_{FV}(x_{m})=\left[0,\ldots ,0,\underset{2D\;\text{non-zero}\;\dim}{\underset{︸}{\Phi_{k}^{\left(1\right)},\Phi_{k}^{\left(2\right)}}},0,\ldots ,0\right].$$ 10

The dimension of FV is higher than traditional BoVW method, and hence PCA [17, 20] is usually applied to reduce the dimension of feature vector which leads to shorter processing time. Since the uncorrelated features and GMM covariance matrices of diagonal assumption are consistent, PCA whitening is also applied to ensure that assumption of diagonal covariance matrix is satisfied. In our system, a total of 128 feature vector is generated per image pixel, which is reduced to 64 using PCA (note that the feature dimension after PCA is empirically set based on our dataset). For further detail of the dimensionality reduction procedure, the readers can refer to the supplementary information.

### SVM Classifier Based on SDCA Method and Learning

SVM has been extensively used in recognition algorithms to find global optimal solution using statistical learning theory and structure risk minimization principle. The main strength of SVM is that it can handle large dimensional data, and thus SVM is used to perform the recognition of the FFSP. A one-versus-rest scoring scheme is implemented to recognize different planes in the US image database. The scoring function for the hyperplane *H* in SVM classifier is defined as:

$$H:w^{T}x_{i}^{}+b=0,\;\;i=1,2,\ldots ,n,$$ 11

where *x*_1_,*x*_2_,…,*x*_*n*_ are input vectors in *R* ^*D*^, *b* ∊ *R* is a bias parameter, *w*_1_,*w*_2_,…,*w*_*n*_ are weighting vectors, and *T* denotes the transpose operator. The main objective of SVM is to obtain optimal *w*_1_,*w*_2_,…,*w*_*n*_ values. SDCA is explored to obtain these optimal values since it is able to achieve high accuracy by dual-prime objectives. Meanwhile, the objective function in SVM can be minimized by SDCA with different loss functions. Given the labels *y*_1_,…,*y*_*n*_ in {±1}, the SVM problem with linear kernel and without bias term is denoted as:

$$\Psi_{i}(\alpha )=max\{0,1-y_{i}\alpha \}.$$ 12

This problem can be converted to solve $\min_{w\in R^{d}}P(w)$, where:

$$P(w)=\left[\frac{1}{n}\sum_{i=1}^{n}\Psi_{i}(w^{T}x_{i})+\frac{\lambda }{2}{\left\|w\right\|}^{2}\right].$$ 13

In our work, the dual problem in Eq (16) is solved by SDCA:

$$\underset{\alpha \in R^{n}}{\max(\Omega (\alpha ))},\text{where}\;\;\Omega (\alpha )=\left[\frac{1}{n}\sum_{i=1}^{n}-\Psi_{{}_{i}}^{*}(-\alpha_{i})-\frac{\lambda }{2}{\left\|\frac{1}{\lambda n}\sum_{i=1}^{n}\alpha_{i}x_{i}\right\|}^{2}\right].$$ 14

Let $w(\alpha )=\frac{1}{\lambda n}\sum_{i=1}^{n}\alpha_{i}x_{i}$, and initialize at: *w* ^(0)^ = *w*(*α* ^(0)^), the objective function at each iteration *t* is obtained by:

$$-\Psi_{{}_{i}}^{*}(-(\alpha_{i}{}^{\left(t-1\right)}+\Delta \alpha_{i}))-\frac{\lambda n}{2}{\left\|w^{\left(t-1\right)}+\frac{1}{\lambda n}\Delta \alpha_{i}x_{i}\right\|}^{2}.$$ 15

The updated rule is as follows:

$$\alpha^{\left(t\right)}\leftarrow \alpha^{\left(t-1\right)}+\Delta \alpha_{i}e_{i}.$$ 16

$$w^{\left(t\right)}\leftarrow w^{\left(t-1\right)}+{\left(\lambda n\right)}^{-1}\Delta \alpha_{i}x_{i}.$$ 17

The final outputs are obtained by averaging the output of *α* and *w* obtained from SDCA method. For the theoretical analysis of this approach, the interested readers can refer to [32] for more detail.

## Experimental Results

### Experiment Setup

**Fig 6:**
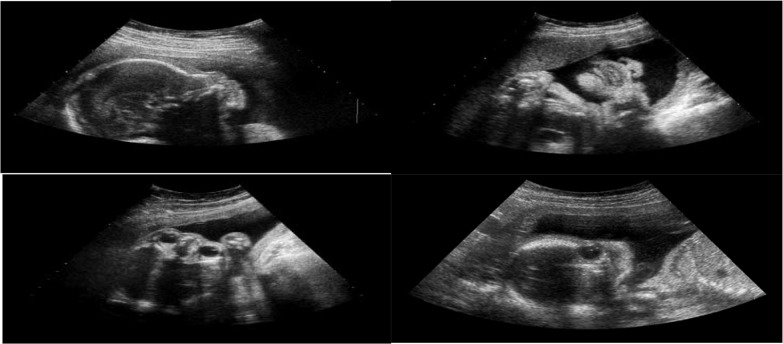
Different planes of fetal ultrasound (US) image samples for training (left upper is axial plane, right upper is coronal plane, left bottom is sagittal plane, and right bottom is non-fetal facial standard plane (FFSP)).

The experimental dataset is composed of 187 images of axial plane, 192 images of coronal plane, 203 images of sagittal plane, and 1153 images without any FFSPs (non-FFSP). All images were extracted from US videos acquired by a commercial US scanner (Acuson Sequoia 512, Siemens Medical Solutions, USA) from Shenzhen Maternal and Child Health Hospital. Fetal gestational age is between 20 and 36 weeks. Conventional US sweep was performed to obtain the videos of pregnant women in the supine position by a radiologist with more than five years of experience in US obstetrics. The typical training samples are shown in Fig 6. Our system was implemented by the mixed programming technology of Matlab and C++. The feature extraction time for an image (size: 576×768) is 0.59 seconds (32GBs RAM, double quad-core multithreaded server with a single CPU). The whole processing time for the testing step requires less than 1 second on a single CPU core (in the case of 2 pixel SIFT density). In our experiments, linear denotes no kernel is applied, whereas Chi2 and Hel. denote the corresponding kernels, respectively. It should be noted that all fetal images in this experiment have informed written consent from all subjects, and the study protocol was reviewed and approved by the Ethics Committee of Shenzhen University.

The FFSP recognition problem is quantitatively evaluated by the recognition accuracy (the ratio between the number of correctly classified samples and the actual number of samples for each class). Quantitatively expressed recognition metrics such as mean average precision (mAP), average accuracy, false positive rate (FPR) (how many images in this class are classified in the other classes), and false negative rate (FNR) (misclassifying a FFSP image as a non-standard one) are also employed to evaluate the FFSP recognition system. Without loss of generality, the experimental results are based on multilayer network using feature hierarchy information. The experiments are repeated at least 10 times and the average results are reported. Our experiments for the proposed system are designed to answer the following questions:

1. Can the SDCA, FV, and normalization method improve the FFSP recognition results based on the dense RootSIFT features?
2. Is it possible to improve the FFSP recognition result using normalization and discriminative learning?
3. Does deep FV based on feature hierarchy improve the FFSP recognition result by multilayer design?

### Effect of Stochastic Dual Coordinate Ascent

The comparison of SDCA and the traditional SGD method based on different feature encoding methods is shown in Table 1. Note that the testing time in Table 1 does not include feature extraction time. This table reveals that the SDCA method has achieved comparable or better performance than the traditional SGD method, which is consistent with the analysis reported in [32]. It also indicates that the dual-primal objective in SDCA is helpful to improve the recognition performance. From these observations, we infer that the aggregating vectors improve accuracy and mAP results. As illustrated in Table 1, the result of FV outperforms BoVW and VLAD algorithms in terms of mAP and accuracy results, but the computational time in training using FV method is higher than BoVW and VLAD methods. It is also found that Chi2 kernel is the most effective kernel methods among the three selected methods for the FFSP recogntion. One possible explanation for these results is that SDCA obtains more optimal SVM solutions using the dual-prime objective than the prime objective in SGD.

**Table 1:** Accuracy, mean average precision (mAP), training and testing time comparison of stochastic gradient decent (SGD) and stochastic dual coordinate ascent (SDCA) method in terms of bag of visual word (BoVW), vector of locally aggregated descriptor (VLAD), Fisher vector (FV) using linear, hell (Hel.) and chi2 methods.

Algorithms	Kernels	Methods	Accuracy	mAP	Training Time(S)	TestingTime (S)
BoVW	Linear	SGD	0.7100	0.9305	157.6702	0.0024
		SDCA	0.8820	0.9922	140.5053	0.0018
	Hel.	SGD	0.8140	0.9473	152.2601	0.0019
		SDCA	0.8923	0.9928	152.0241	0.0018
	Chi2	SGD	0.8425	0.9829	444.2517	0.0021
		SDCA	0.9054	0.9931	358.5726	0.0020
VLAD	Linear	SGD	0.8419	0.9894	670.0718	0.0022
		SDCA	0.8938	0.9937	360.7802	0.0015
	Hel.	SGD	0.8287	0.9873	592.0701	0.0024
		SDCA	0.8813	0.9928	337.4783	0.0015
	Chi2	SGD	0.8419	0.9862	1845.1721	0.0021
		SDCA	0.8813	0.9928	1118.8032	0.0020
FV	Linear	SGD	0.9077	0.9933	1321.0592	0.0020
		SDCA	0.9224	0.9907	778.1201	0.0017
	Hel.	SGD	0.8938	0.9931	1060.1001	0.0021
		SDCA	0.9207	0.9919	612.6392	0.0017
	Chi2	SGD	0.8813	0.9940	3161.5600	0.0022
		SDCA	0.9320	0.9919	2031.5000	0.0019

The training time for the FFSP data is shown in Table 1. SDCA outperforms SGD in terms of training time. Due to offline training, the constraint of the realtime application is often determined by testing rather than training. SDCA significantly improves the computational efficiency of the SVM classifier, and it is observed that less than 1s in testing is taken for FFSP even with Chi2 kernel. This observation indicates that the FFSP system is suitable for realtime application. Apart from the better performance achieved by SDCA than SGD, the processing time of SDCA is shorter than SGD as shown in Table 1. The reason is that SDCA has a clear stopping condition with the duality gap calculation, and hence SDCA is more efficient than SGD. Furthermore, SDCA is a generic principle applicable to many algorithms and simple to implement. Although other on-line learning algorithms are closely related to SDCA (i.e., passive- aggressive algorithm), the fast convergence rule makes SDCA a desirable and promising solution for the FFSP learning and recognition.

Effect of Multilayer Fisher Network

**Fig 7:**
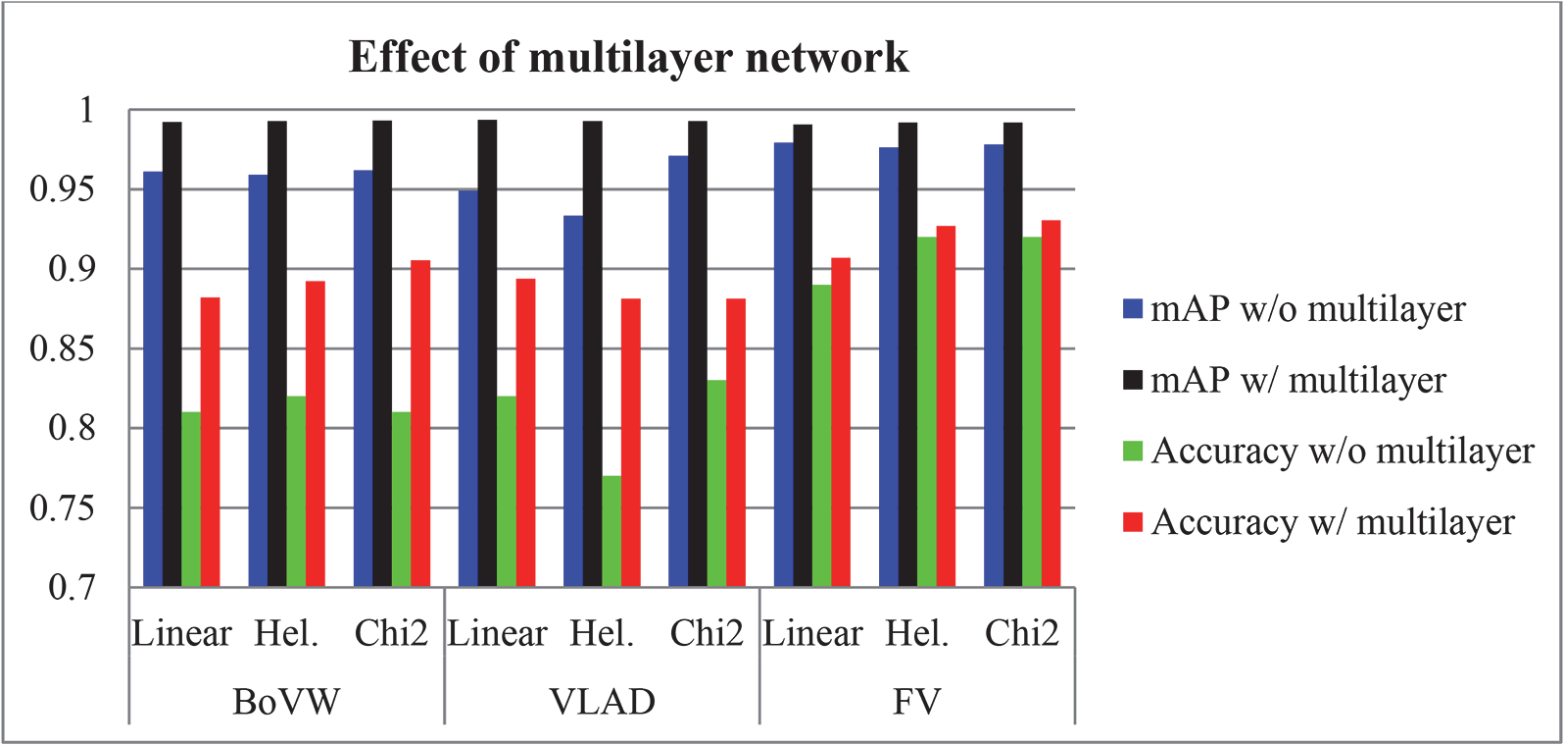
Effect of with and without multiple layer using bag of visual word(BoVW), vector of locally aggreaged descriptor (VLAD) and Fisher vector (FV) encoding method with liner, hell (Hel.) and chi2 kernel algorithm.

**Fig 8:**
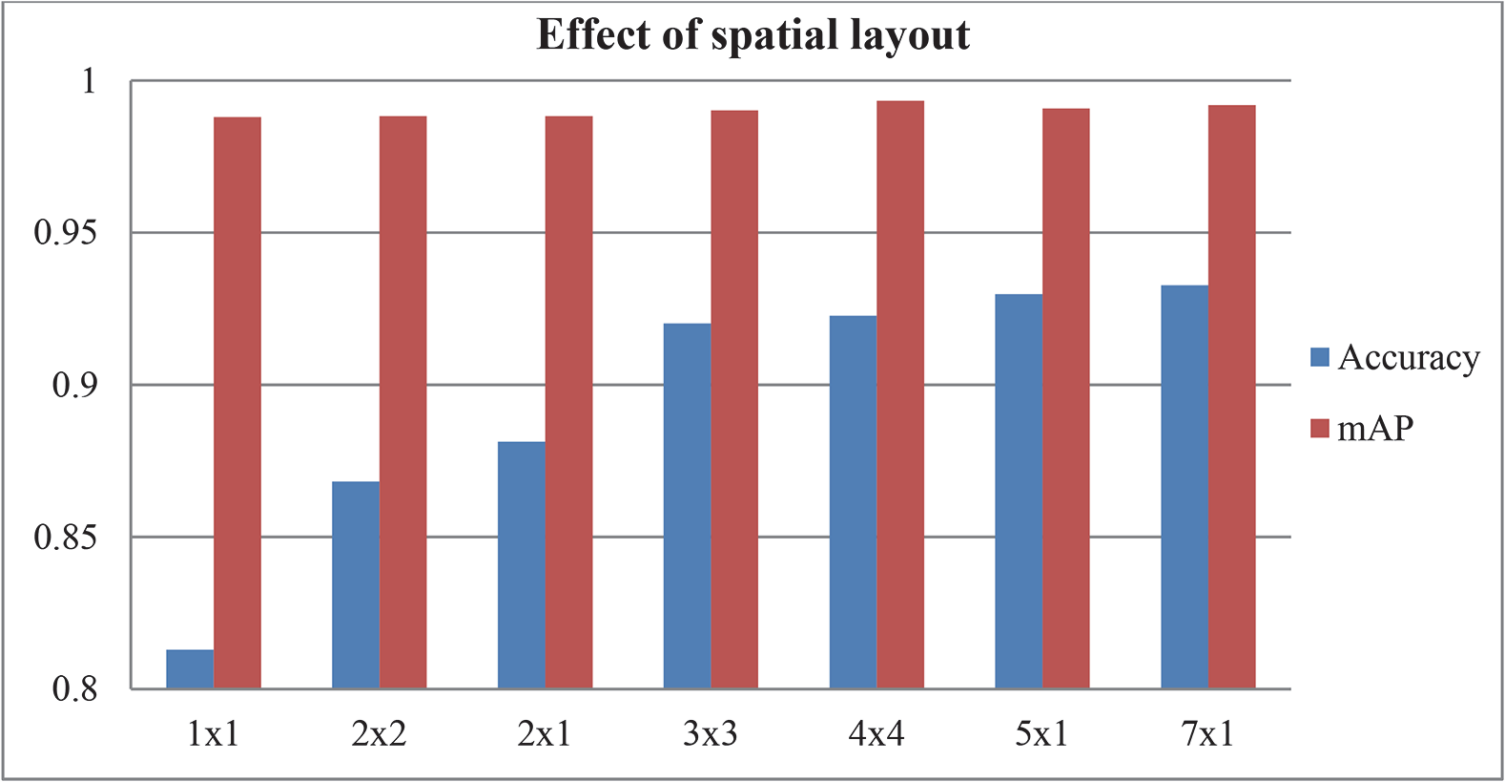
Effect of different kinds of spatial layout for multilayer network of Fisher vector (FV).

To demonstrate the effectiveness of feature hierarchy with multilayer architecture design, the FFSP recognition is performed on different spatial stacking methods. Fig 7 shows the recognition results with and without feature hierarchy by multilayer network architecture. It is found that deep FV by feature hierarchy improves both accuracy and mAP results. These results validate the theoretical assertion and generalization ability of spatial pyramid model [25]. Fig 8 shows the effect of selected spatial stacking layout. It is revealed that the dense RootSIFT feature and spatial stacking could be integrated to improve the recognition performance as well. However, it can be seen that the accuracy and mAP do not infinitely increase with the increase of partitioning regions in the image. This is due to the fact that overlapping and unrelated information increases as more regions are generated by spatial stacking and such information does not increase the recognition performance.

### Effect of Normalization

**Fig 9:**
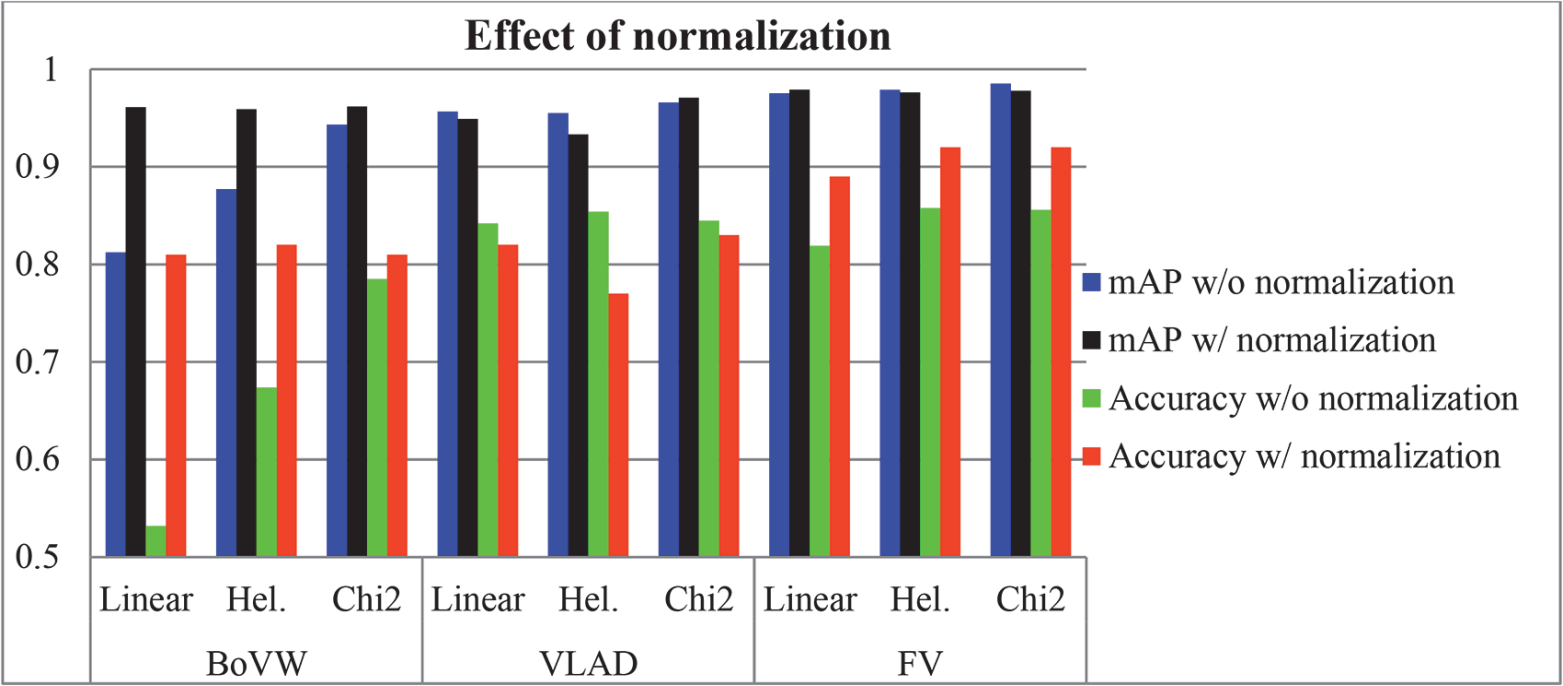
Effect of with and without normalization methods with bag of visual word (BoVW), vector of locally aggreaged descriptor (VLAD) and Fisher vector (FV) using liner, hell (Hel.) and chi2 kernel algorithm.

It is reported in [19] that normalization methods can be integrated with different homogeneous kernels to further improve recognition accuracy. In this sub-section, experiments are conducted to validate the effects of several normalization methods. As shown in Fig 9, higher recognition accuracy and mAP are obtained with normalization methods, which is in agreement with the findings of normalization method reported in [19]. It can be seen that the proposed normalization method substantially improves recognition result. In fact, normalization method is found to be a highly effective approach to improve FFSP recognition accuracy when many variations are found in each class. There are three key reasons for this improvement. First, normalization method is effective as it removes the background information. Second, power normalization reduces the bursty effect. Third, histogram stretching or normalization expands the dynamic range of the code words and improves the recognition effectiveness.

### Effect of Different Portion of Training Images

**Fig 10:**
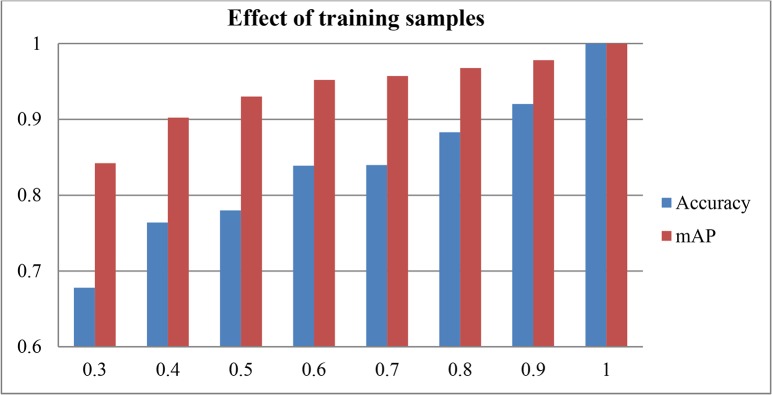
Accuracy and mean average precision (mAP) results of different portion of training samples.

**Fig 11:**
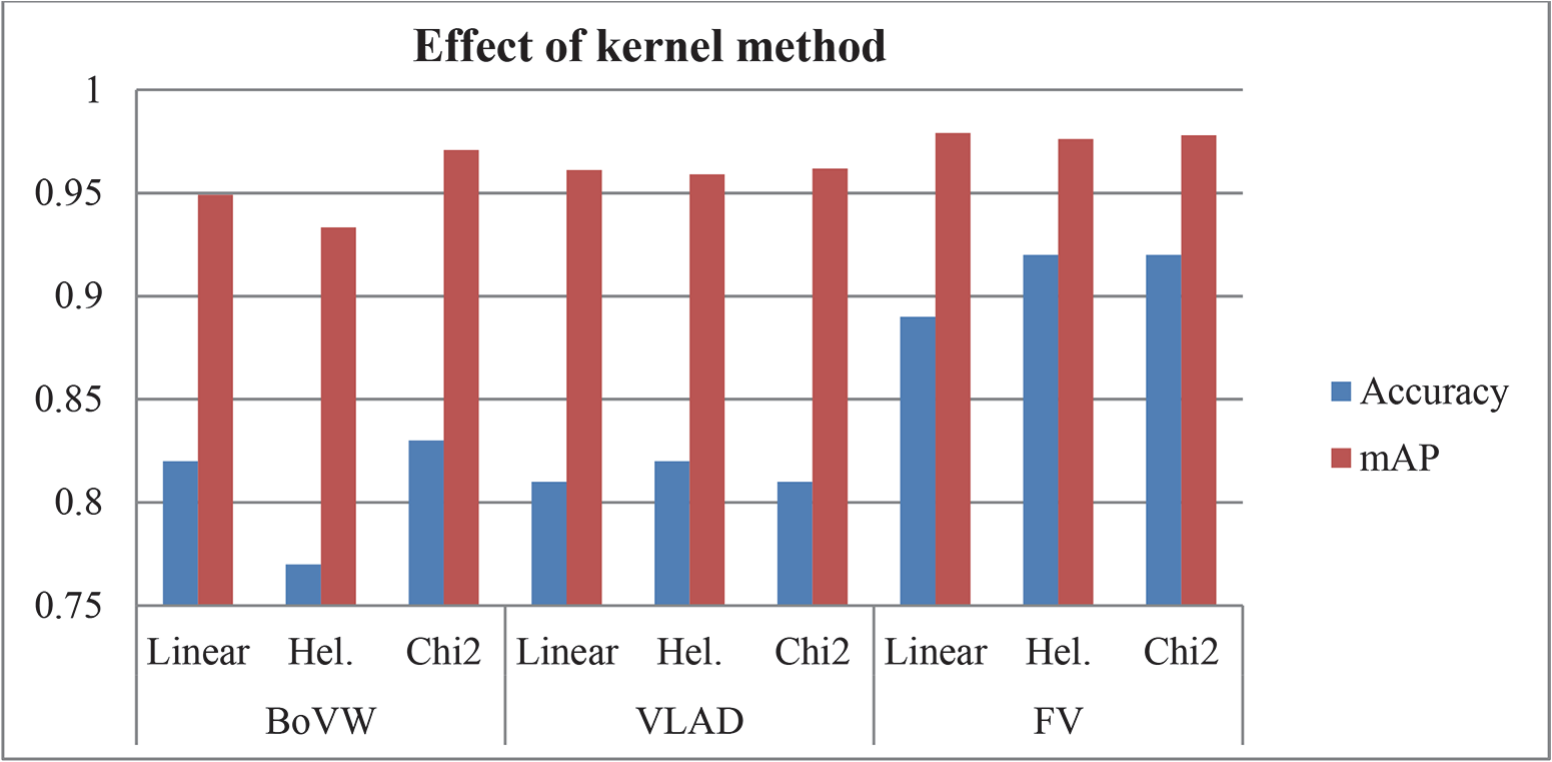
Accuracy and mean average precision (mAP) results of linear, hell (Hel.) and chi2 kernel methods with bag of visual word (BoVW), vector of locally aggregated descriptor (VLAD) and Fisher vector (FV).

The effect of the number of the training images is tested as one of the performance indices since availability of training images is usually limited in most situation. In our experiment, a certain portion of images are randomly selected as training images and the remaining images are used as testing images. The learnt parameters from the training images are then used on the testing images. This process is repeated several times with new samples randomly selected from the training images. Fig 10 shows accuracy and mAP for different percentage of training images, where percentage 1 means that only one image is used for testing and the remaining images are adopted for training in each class (leave one out method). As seen from Fig 11, with the increase of training images, the performance is increased monotonically. The accuracy and mAP are 0.9327 and 0.9919 using 90% percent of image for training, respectively, which demonstrates 90% images for training is suitable for FFSP.

### Recognition Results

As discussed in [19], better recognition results are obtained by calculating an explicit feature map that approximates a non-linear kernel (without feature map) as a linear one. In other words, this approach produces feature vectors by non-linear mapping. Feature mapping is derived from the popular kernel trick method and can be applied to any approaches based on the distance metric (i.e., nearest neighbors). For this mapping, the Chi2 kernel achieves the best results among the other kernel methods.

**Fig 12:**
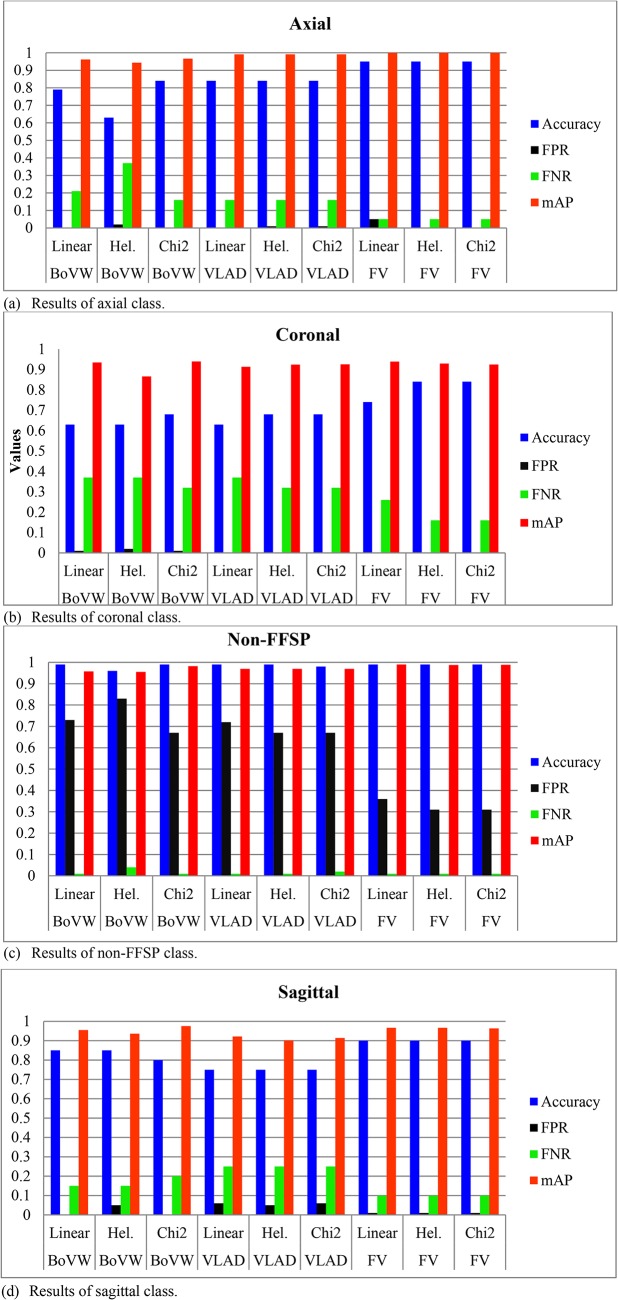
Accuracy, false positive rate (FPR), false negative rate (FNR) and mean average precision (mAP) results of bag visual word (BoVW), vector of locally aggregated descriptor (VLAD), Fisher vector (FV) using linear, hell (Hel.), and chi2 method of (a) axial class (b) coronal class (c) non-fetal facial stand plane (non-FFSP) class (d) sagittal class.

Fig 12 shows the recognition results for the four classes in terms of accuracy, FPR, FNR, and mAP results. It can be seen that mAP is generally higher than accuracy among the four classes. It is also evident that FPR and FNR results are quite low except for non-FFSP class. From Fig 12, it can be observed that FV algorithm significantly outperforms VLAD and BoVW in terms of FPR and FNR. The preliminary explanation of superior performance from FV algortihm over VLAD and BoVW is that FV has soft assignment and high order statistics to enhance the discriminabity of different classes. Apart from the above observations, sagittal and coronal planes are generally easier to discriminate than the axial plane since better recognition is achieved in both sagittal and coronal planes than the axial plane. Moreover, it is noteworthy that the FPR result in the non-FFSP class is quite high, which is probably caused by high correlation between images of FFSPs and the non-standard plane extracted from the video sequence. In each video sequence, all images are highly correlated with each other when they are from neighboring frames. Overall, both FPR and FNR results in the standard planes are very low, which validate the effectiveness of the proposed FFSP recognition system.

The comprehensive comparison results indicate that high recognition results are obtained in each class using FV encoding method. Generally, aggregating vectors methods (VLAD and FV) outperform the traditional BoVW method. In addition, spatial layout model is able to improve the recognition performance by making use of the spatial structure information. In addition, FV algorithm obtains the best recognition performance among all algorithms. High accuracy is an important indicator for practical application in clinical practice, and hence FV is quite suitable for the FFSP recognition.

**Fig 13:**
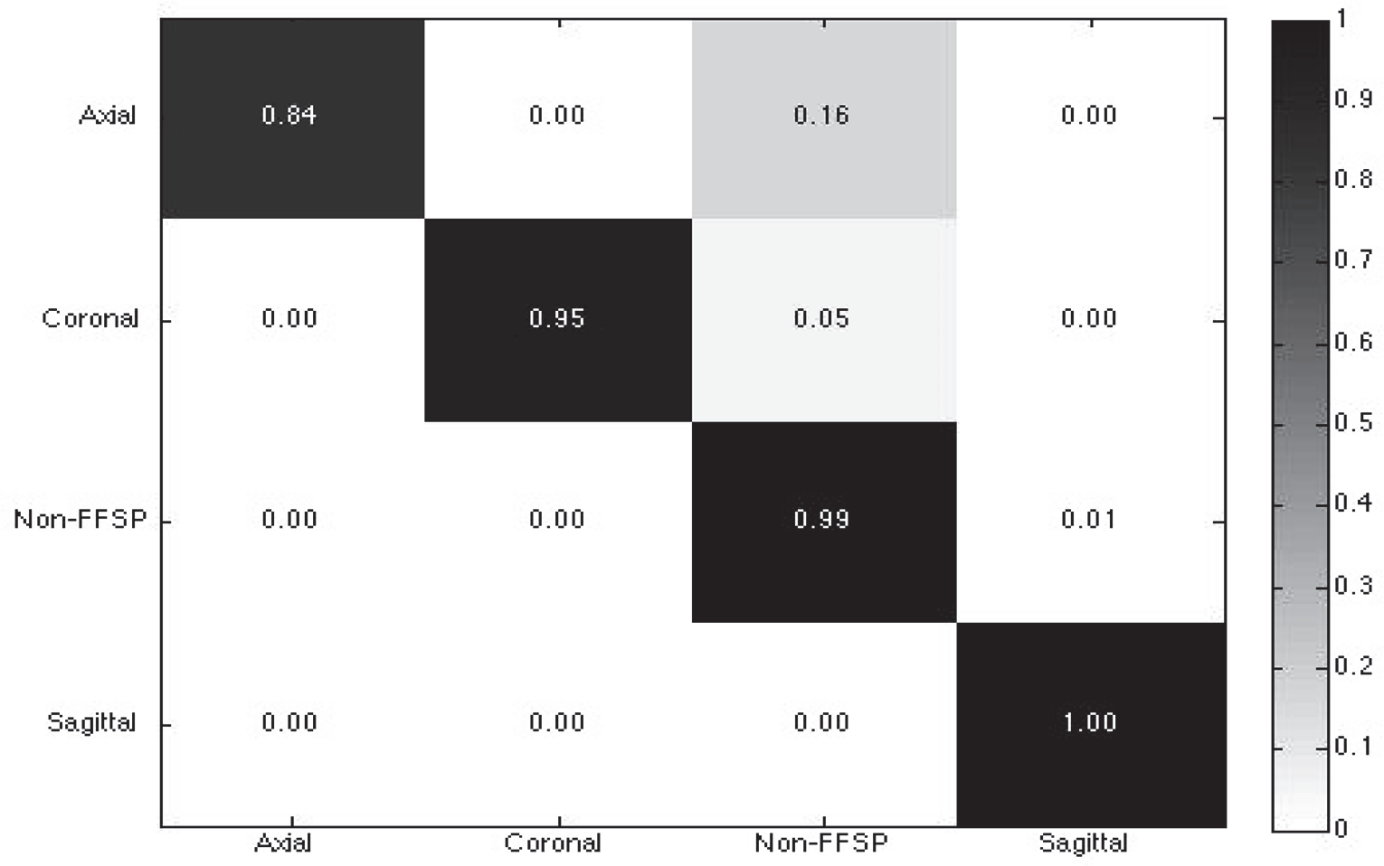
Confusion matrix for fetal facial standard plane (FFSP) recognition.

**Fig 14:**
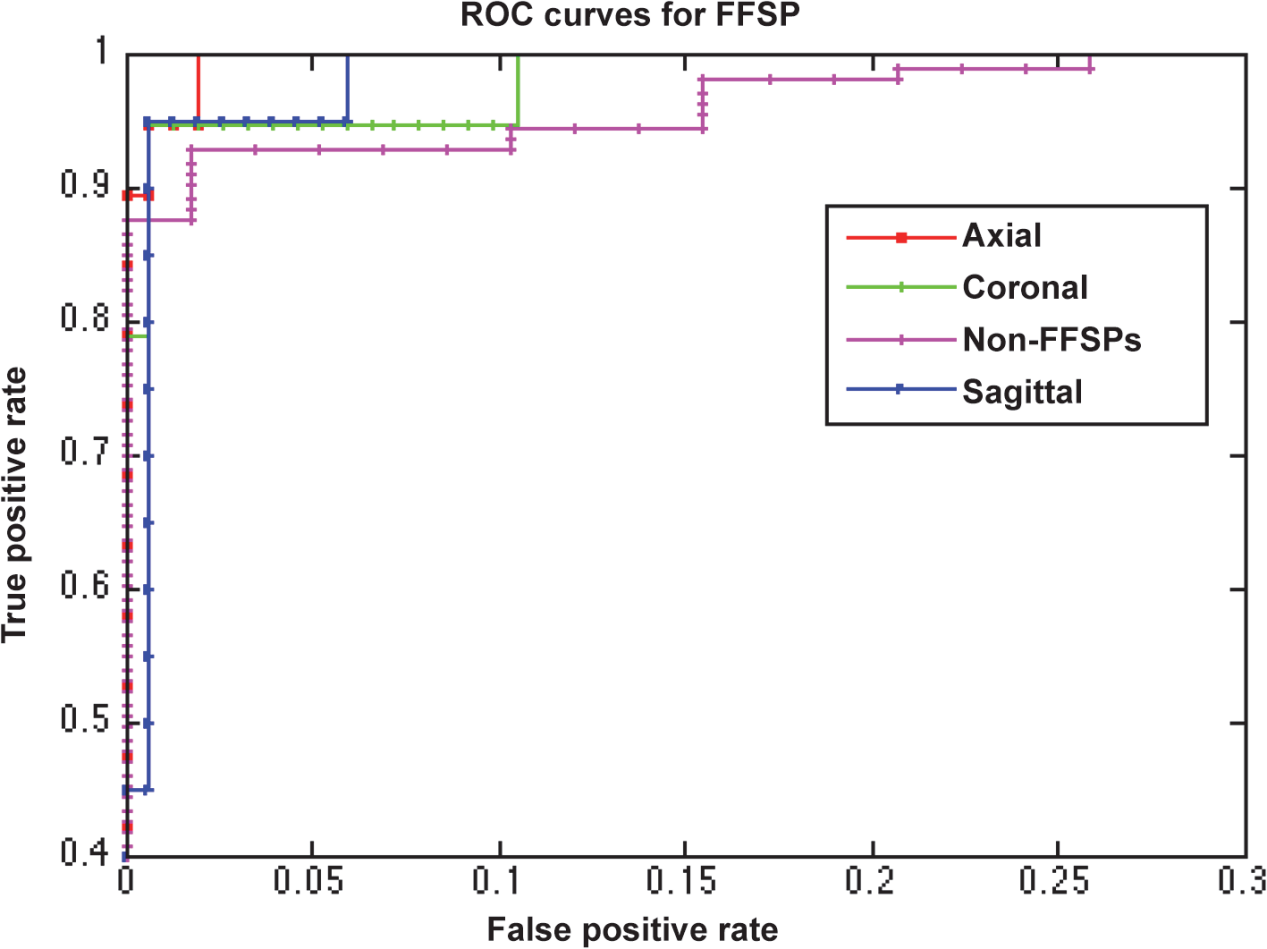
Receiver operation characteristics (ROC) curves of fetal facial standard plane (FFSP) recognition.

The confusion matrix of the FFSP recognition is shown in Fig 13. The rows and columns represent the actual FFSP labels and predicted labels, respectively. The diagonal elements represent the mean recognition accuracy for each class. As seen from the confusion matrix, the overall recognition accuracy for each class is very high and the mis-recognition ratio is very low. Fig 14 shows the ROC curves for FFSP recognition, which further confirms the proposed method is effective to recognize axial, coronal, and sagittal view of the ultrasound images.

**Fig 15:**
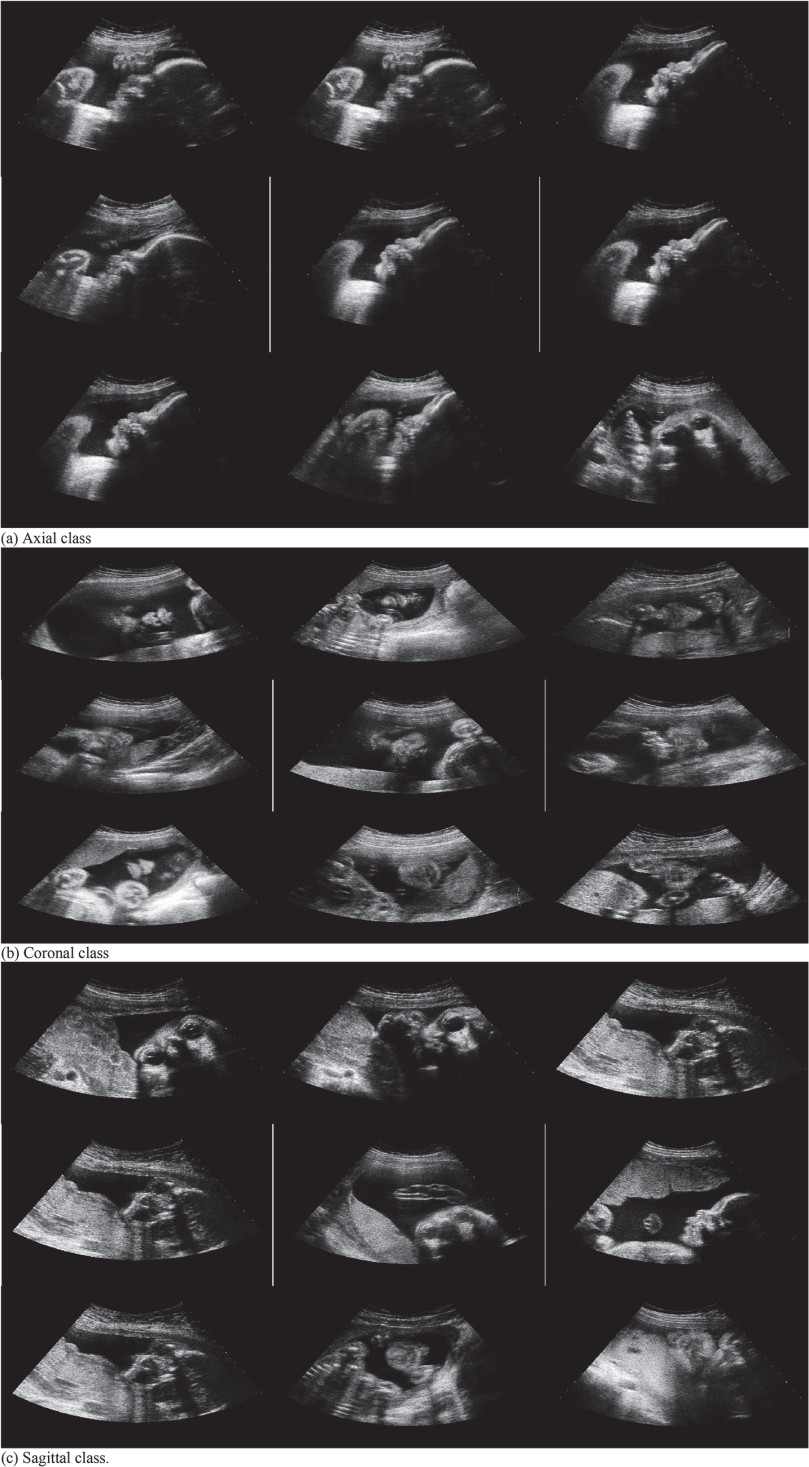
Top similar ultrasound images of the input sample images of (a) axial class (b) coronal class (c) sagittal class. The first one is the input query images and the remaining images are retrieved based on the similarity with the query image.

To validate the effectiveness of the feature representation for the FFSP recognition, we examine the features of similar ultrasound images (see Fig 15) of axial, coronal and sagittal classes. The top left image is the input FFSP plane image for the similarity test, and the rest are the ranked images (from left to right, top down) based on the similarity score of the first image. The visual similarity of the axial, coronal, and sagittal classes clearly validates the extracted feature is highly discriminative. Overall, the visual similarity based on the ranked scores confirms the proposed method is capable of recognizing the axial, coronal, and sagittal views of the ultrasound images.

## Discussions

An automatic solution for recognition of FFSP in US images based on RootSIFT, FV with multilayer design, and SDCA is presented in this paper. The experimental results show that the proposed method successfully recognizes the important FFSPs with high performance. Moreover, the methodologies utilized in this work can be extended to other fields to classify and detect standard planes in other organs (i.e., abdomen, breast, prostate, lung and liver), as well as to prediction and recognition of cancerous cells. Other advanced techniques can be used to further improve the recognition performance of the proposed algorithm in our future work. For example, hierarchical fusion of the dense and sparse features would be very beneficial for the FFSP recognition. Apart from US image information, more modality information such as MRI and CT can be adopted. Furthermore, fusion of classifiers should be interesting for FFSP recognition. For instance, unsupervised neural network based on deep learning can be integrated with supervised SVM classifier to further improve its performance. Last but not least, segmentation and prediction algorithms can also be explored.

## Supporting Information

S1 File Dimensionality Reduction. (DOCX)

S2 File Feature Normalization. (DOCX)
