# Supplementary material for: Automatic Recognition of Fetal Facial Standard Plane in Ultrasound Image via Fisher Vector
Source: PLoS One. 2015 May 1;10(5):e0121838. doi: 10.1371/journal.pone.0121838 (PMC4416891; doi:10.1371/journal.pone.0121838)
Supplement: S2 File — (DOCX) [file pone.0121838.s002.docx]

**Appendix S2 Feature Normalization**

Given the variability of information in the training dataset, normalizing the feature is shown to be very effective to improve the recognition. Feature normalization by component-wise $l_{p}$ norm is an important step to enhance performance because the effect of background information in images is removed. By dividing each matrix with the corresponding norm, $l_{p}$ norm distributes the feature matrices evenly around a hypersphere with radius of 1. $l_{p}$ norm on real number when$p\geq1$is defined as:

$\left\|x\right\|_{p}=\left(\sum_{i=1}^{n} |x_{\mathrm{i}}|^{p}\right)^{1/p},$ (B.1)

where $p=1$is $l_{1}$norm, and $p=2$ is $l_{2}$norm or the Euclidean norm.

Assuming a given dataset, $u_{i}\sim\mathrm{U}(p,n)$ follows a distribution *p* using the *i*.*i*.*d* model and the feature matrix is uniformly distributed in an unit sphere of an *n* dimensional space, the closed form solution to the normalized coordinates: $u_{i}=\frac{x_{i}}{||x||_{p}}$, is denoted as:

$\psi_{p,n}(u_{i})=\frac{p\Gamma(n/p)}{2\Gamma(1/p)\Gamma((n-1)/p)}(1-|u_{i}|^{p})^{(n-1)/p-1},u_{i}\in[-1,1],$ (B.2)

where $\Gamma(\bullet)$is the gamma function. It is noted that Eq. (6) is a Gaussian distribution for$p=2$. Burrascano found that the $l_{p}$metric is a good measure between data points if the data is distributed in a generalized Gaussian. The $l_{p}$ norm can be formulated as finding the one with the maximum entropy among all pdf of$f_{p}$by the maximum spreading:

$f_{p}(x)=\frac{p(1-1/p)}{2\Gamma(1/p)(\sigma_{p})^{p}}\exp\left(1-\frac{|x-x_{0}|^{p}}{p(\sigma_{p})^{p}}\right),$ (B.3)

where$(\sigma_{p})^{p}=constant$ and $(\sigma_{p})^{p}=1.$ To reduce the variability across the space dimension, scaling the features with their non-Gaussian magnitudes is performed to render the features more Gaussian-like. $l_{2}$norm approximately removes the content-independent information (background) .

In general, a few artificially large components affect the similarity scores of two matrices in SVM by dominating the computed similarity. Such bursty component tends to lead to a suboptimal measure of the similarity score. Therefore, the large values in the feature matrix corresponding to such component should be suppressed. A simple yet effective method such as power normalization is employed and denoted as:

$z\leftarrow sign(\mathrm{z})|\mathrm{z}|^{\rho},0\leq\rho\leq1.$ (B.4)

In our experiment, $\rho$is set: $\rho=0.5$, which is the signed square rooting or more simply square rooting. Performing power normalization can ‘‘unsparsify’’ data to reduce influence of bursty features. Besides, power normalization is regarded as an explicit data representation of the Hel. kernel. It is noted that power normalization by square-root transform is not specific and beneficial to the FFSP recognition.
